# The histone methyltransferase WHSC1 is regulated by EZH2 and is important for ovarian clear cell carcinoma cell proliferation

**DOI:** 10.1186/s12885-019-5638-9

**Published:** 2019-05-15

**Authors:** Machiko Kojima, Kenbun Sone, Katsutoshi Oda, Ryuji Hamamoto, Syuzo Kaneko, Shinya Oki, Asako Kukita, Hidenori Machino, Harunori Honjoh, Yoshiko Kawata, Tomoko Kashiyama, Kayo Asada, Michihiro Tanikawa, Mayuyo Mori-Uchino, Tetsushi Tsuruga, Kazunori Nagasaka, Yoko Matsumoto, Osamu Wada-Hiraike, Yutaka Osuga, Tomoyuki Fujii

**Affiliations:** 1 Department of Obstetrics and Gynecology, Graduate School of Medicine, The University of Tokyo, 7-3-1 Hongo Bunkyo-ku, Tokyo, 113-8655 Japan; 2 Division of Molecular Modification and Cancer Biology, National Cancer Center Research Institute, 5-1-1 Tsukiji, Chuo-ku, Tokyo, 104-0045 Japan; 3 Department of Obstetrics and Gynecology, Teikyo University School of Medicine, 2-11-1, Kaga, Itabashi-ku, Tokyo, 173 0003 Japan

**Keywords:** Histone methyltransferase, Wolf-Hirschhorn syndrome candidate gene-1, Enhancer of zeste homolog 2, Ovarian clear cell carcinoma, Epigenetic modifier, EZH2 selective inhibitor, H3K36 dimethylation, cell proliferation

## Abstract

**Background:**

Wolf-Hirschhorn syndrome candidate gene-1 (WHSC1), a histone methyltransferase, has been found to be upregulated and its expression to be correlated with expression of enhancer of zeste homolog 2 (EZH2) in several cancers. In this study, we evaluated the role of WHSC1 and its therapeutic significance in ovarian clear cell carcinoma (OCCC).

**Methods:**

First, we analyzed *WHSC1* expression by quantitative PCR and immunohistochemistry using 23 clinical OCCC specimens. Second, the involvement of WHSC1 in OCCC cell proliferation was evaluated by MTT assays after siRNA-mediated WHSC1 knockdown. We also performed flow cytometry (FACS) to address the effect of WHSC1 on cell cycle. To examine the functional relationship between EZH2 and WHSC1, we knocked down EZH2 using siRNAs and checked the expression levels of WHSC1 and its histone mark H3K36m2 in OCCC cell lines. Finally, we checked *WHSC1* expression after treatment with the selective inhibitor, GSK126.

**Results:**

Both quantitative PCR and immunohistochemical analysis revealed that *WHSC1* was significantly overexpressed in OCCC tissues compared with that in normal ovarian tissues. MTT assay revealed that knockdown of WHSC1 suppressed cell proliferation, and H3K36me2 levels were found to be decreased in immunoblotting. FACS revealed that WHSC1 knockdown affected the cell cycle. We also confirmed that WHSC1 expression was suppressed by EZH2 knockdown or inhibition, indicating that EZH2 is upstream of WHSC1 in OCCC cells*.*

**Conclusions:**

WHSC1 overexpression induced cell growth and its expression is, at least in part, regulated by EZH2. Further functional analysis will reveal whether WHSC1 is a promising therapeutic target for OCCC.

**Electronic supplementary material:**

The online version of this article (10.1186/s12885-019-5638-9) contains supplementary material, which is available to authorized users.

## Background

Ovarian clear cell carcinoma (OCCC) was defined by the World Health Organization as one of the histologic subtypes of ovarian cancer in 1973. The recent surveillance and epidemiology showed that the incidence of OCCC in the United States is 4.8% in Caucasians, 3.1% in African American, and 11.1% in Asians. In Japan, the number of cases of OCCC is about 25% that of epithelial ovarian cancers, which is higher than that in western countries [1]. OCCC is known to be resistant to platinum-based, front-line chemotherapy and shows a worse prognosis compared with serous carcinoma or endometrioid carcinoma [1–3]. The most frequent and important genetic alterations observed in OCCC are those that occur in the chromatin remodeling factor gene, AT rich interactive domain 1A (*ARID1A*). It has been reported that over 50% of OCCC patients have mutations in *ARID1A* [4].

Histone methylation is one of the important epigenetic modifications, along with histone acetylation, phosphorylation, ubiquitination, poly ADP-ribosylation, and sumoylation, and is generally associated with gene expression [5]. Many reports have suggested a role of histone methylation dysregulation in carcinogenesis and cancer progression [6]. In addition, several types of histone methyltransferases have been reported to play important roles in tumor progression in many types of cancers [7]. For instance, our previous study showed that SUV39H2, a the histone methyltransferase caused therapeutic resistance in cancer cells [8].

Wolf-Hirschhorn syndrome candidate 1 (WHSC1) is a SET-domain containing histone methyltransferase [9]. To activate transcription in various regions of the genome, WHSC1 specifically catalyzes the dimethylation of lysine 36 of histone H3 (H3K36me2), a histone mark associated with the open chromatin region [10]. Although recent reports suggest that WHSC1 is overexpressed in multiple solid cancers [11, 12], there are no reports regarding its expression profile and function in OCCC.

Enhancer of zeste homolog 2 (EZH2) is one of the most widely studied histone methyltransferases in cancer research. EZH2 tri-methylates H3K27 to silence target gene expression. Increased EZH2 activity is known to have an oncogenic effect by repressing tumor suppressor gene expression [13]. Previously, we reported EZH2 overexpression in endometrial cancer cell lines and clinical samples. We also found that knockdown of its expression or the use of an EZH2-selective inhibitor could suppress cell growth and induce apoptosis [14]. In OCCC, it has been reported that EZH2 inhibition has a synthetic lethal effect in *ARID1A*-mutated ovarian cancer cells [15].

The present study was undertaken to elucidate the involvement of WHSC1 in OCCC and to evaluate its potential for therapeutic targeting. To this end, we compared the expression of WHSC1 in clinical OCCC samples and normal ovarian tissues. We further blocked WHSC1 functions in OCCC cells to determine the specific effects on cellular behaviors related to cancer development and progression. In addition, we analyzed the relationship between EZH2 and WHSC1 in OCCC cells. These findings will provide a foundation for further investigation of the roles of histone methylation in carcinogenesis and new effective therapeutic strategies.

## Methods

### Tumor samples

Tumor specimens were acquired from 23 OCCC patients and 3 patients with normal ovaries who underwent surgery at the University of Tokyo Hospital (Additional file 1: Table S1). The specimens were frozen in liquid nitrogen immediately after collection and then stored at − 80 °C until RNA extraction. For the use of specimens in this research, informed consent was obtained from all patients, and the study was approved by the University of Tokyo Genetic Analysis Research Ethics Committee.

### Cell lines and EZH2 inhibitor

OVISE (JCRB1043),OVTOKO (JCRB1048) and RMG-I (JCRB0172) OCCC cell lines, were obtained from the Japanese Collection of Research Bioresources Cell Bank (Ibaraki, Osaka, Japan). OVISE and OVTOKO are ARID1A-mutated cell lines, RMG-Iis ARID1A-wildtype. Both OVISE and OVTOKO were maintained in RPMI1640 medium with 10% fetal bovine serum, RMG-Iwas maintained in Ham’s F12 with 20% FBS. We used the International Cell Line Authentication Committee (ICLAC) database to confirm that these cell lines were not cross-contaminated or misidentified. In addition, we used the MycoAlert™ Mycoplasma Detection Kit (LT07–218, Lonza, Tokyo, Japan) to ensure that there was no mycoplasma contamination before and after the study. EZH2 inhibitor GSK126 was purchased from Active Biochemicals (Maplewood, NJ, USA).

### Quantitative PCR

RNeasy Mini Kit (Qiagen, Valencia, CA, USA) was used for total mRNA extraction according to the manufacturer’s protocol. We used ReverTra Ace (Toyobo, Osaka, Japan) for reverse transcription. *WHSC1* and *EZH2* mRNA levels were measured by quantitative real-time PCR. We designed specific primers for WHSC1, EZH2, and GAPDH (Additional file 1: Table S2). Real-time PCR was performed using the One-Step SYBR PrimeScript RT-PCR Kit (TaKaRa Bio, Tokyo, Japan) in a Light Cycler instrument (Roche, Basel, Switzerland). GAPDH (housekeeping gene) mRNA levels were used for normalization.

### Western blot analysis

After treating the OCCC cells with WHSC1-specific siRNAs or GSK126 for the indicated times at the indicated concentrations, total protein was extracted and transferred to a nitrocellulose membrane as previously described [16, 17]. Primary antibody diluted with blocking buffer was added to the membrane and reacted overnight at 4 °C. The primary antibodies used in this study were anti-WHSC1 (75,359, Abcam, Cambridge, UK), anti-EZH2 (PA0575, Leica Biosystems, Wetzlar, Germany), anti-H3K36me2 (2901, Cell Signaling Technology, Danvers, MA, USA), anti-H3K27me3 (9733, Cell Signaling Technology), and anti-β-actin (Sigma-Aldrich, St. Louis, MO, USA).

### Immunohistochemical staining

The expression patterns of WHSC1 in the OCCC samples and normal ovary specimens were confirmed by immunohistochemistry (IHC) (Additional file 1: Table S3). Briefly, we first performed deparaffinization and rehydration of the paraffin-embedded ovarian carcinoma specimens and normal ovarian tissue slides. Next, we microwaved the slides for 20 min with antigen retrieval buffer (pH 9; S2367, DAKO, Glostrup, Denmark). Anti-WHSC1 antibody (dilution: 1:200; ab75359, Abcam) was added to the tissue sections and incubated overnight at 4 °C. After washing with phosphate buffered saline (PBS), secondary antibody reaction was performed using substrate buffer (K5007, DAKO) and color development reaction with diaminobenzidine (DAB). We then stained the samples briefly with hematoxylin and then covered them with cover slips [18].

### Transfection of OCCC cells with siRNA against WHSC1 or EZH2

OCCC cells were transfected with siRNA (100 nM) against WHSC1 or EZH2 (Additional file 1: Table S4) and with the negative control siRNA (siNC; Sigma Aldrich MISSION siRNA Universal Negative Control SIC-001-25) using Lipofectamine-RNAi MAX transfection reagent (Invitrogen, Carlsbad, CA, USA) for 48–96 h as previously described [19]. At first, the OCCC cells (1 × 10^5^/well) were seeded in 6-well plates for immunoblotting and FACS analyses and in 24-well plates (2 × 10^4^/well) for cell proliferation assay. Next, we incubated the cells for 24 h and treated them with WHSC1-specific siRNAs.

### Cell proliferation assays

Proliferation assays were performed using the WST method. Cells (2 × 10^4^/well) were incubated in 24-well plates before treatment with WHSC1-specific siRNAs. After siRNA treatment for 48–96 h, we added Cell Counting Kit-8 (Dojindo, Tokyo, Japan) reagent to each well, and measured the absorbance at 450 nm, using Epoch™ Microplate Spectrophotometer (BioTek, Winooski, VT, USA) [20].

### Flow cytometry

Cells were fixed in 70% ethanol, and cell cycle analysis was performed by staining with propidium iodide according to standard protocols. DNA content was measured by a.

FACS Calibur HG (Becton Dickinson, Franklin Lakes, NJ, USA) and examined using CellQuest Pro ver. 3.1. (Becton Dickinson).

At first, cells (1 × 10^5^/well) were incubated in 6-well plates and treated with WHSC1-specific siRNAs for 48–72 h. Subsequently, we performed trypsinization, washing with PBS, and fixing with ice-cold 70% ethanol, followed by incubation overnight at 4 °C. After washing the cells with PBS, RNase A (0.25 mg/mL, Sigma-Aldrich) was added and the cells again incubated at 37 °C for 30 min, followed by staining with 50 μg/mL propidium iodide (Sigma-Aldrich) at 4 °C for 30 min in the dark.

### Statistical analysis

Statistical analysis was conducted using JMP Pro. v.14 (SAS, Cary, NC, USA). The correlation coefficient between *WHSC1* and *EZH2* was calculated using the CORREL function. The t-test was used to compare two groups, and one-way analysis of variance (ANOVA) followed by Tukey’s post-hoc test was used to compare three or more groups. *P* < 0.05 was considered to indicate a statistically significant difference.

## Results

### WHSC1 is overexpressed in ovarian clear cell carcinoma cell

**Fig. 1:**
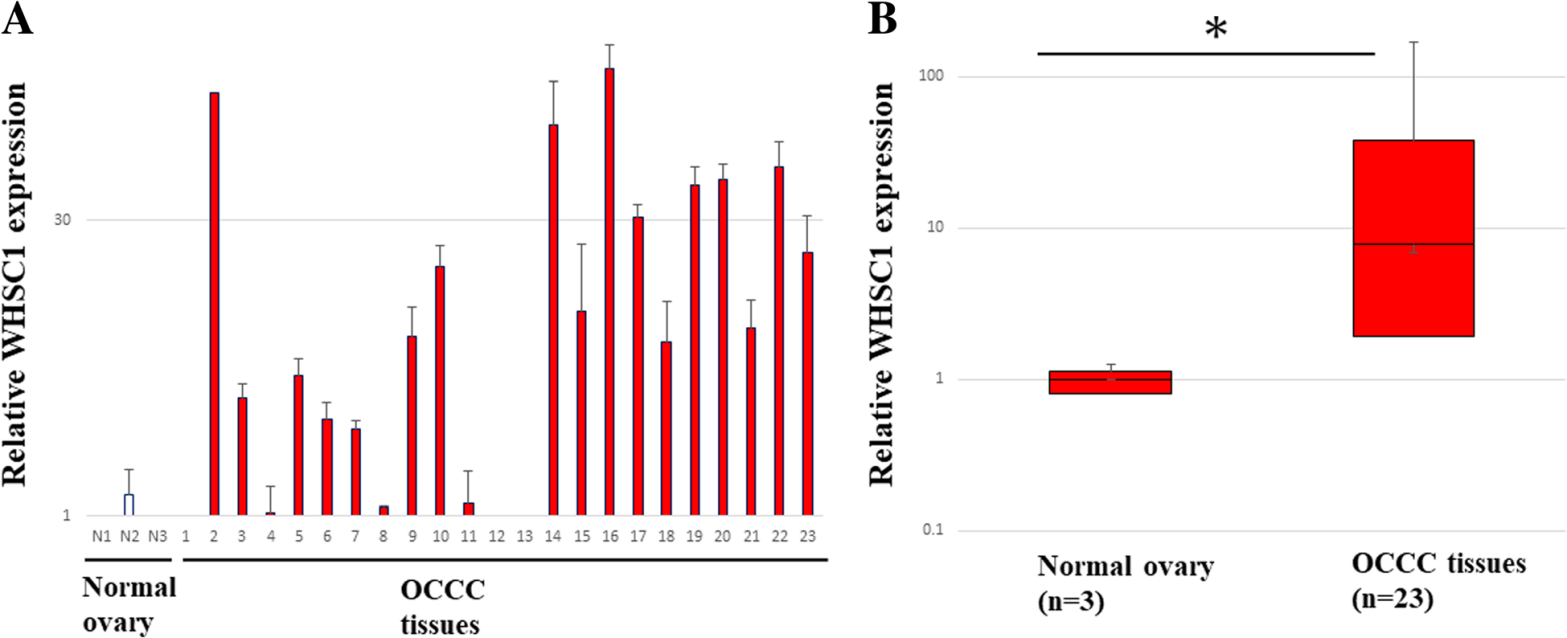
*WHSC1* expression in ovarian clear cell carcinoma (OCCC) and normal ovarian tissue specimens. (**a**) mRNA levels of *WHSC1* were quantitated by real time-qPCR in 23 primary OCCC clinical specimens and three normal ovarian tissues. (**b**) The results are presented as box-whisker plots. The data plotted represent the mean ± standard deviation. (*p < 0.01)

**Fig. 2:**
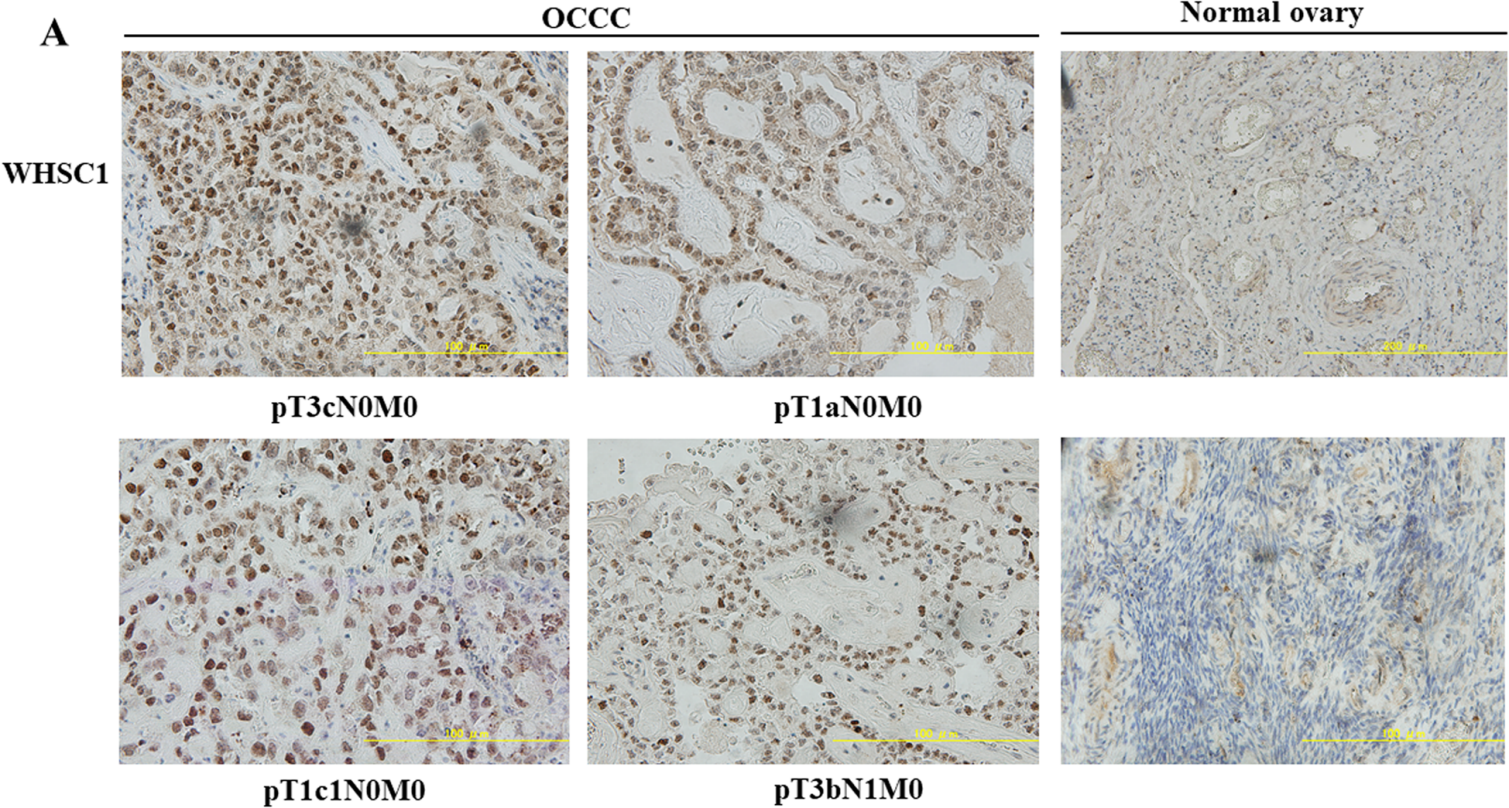
Immunohistochemical staining for WHSC1 expression in OCCC and normal ovary tissues. Clinical information for each section is represented under histologic pictures

First, we analyzed the expression of the histone methyltransferases by RT-PCR (data not shown). We noticed that *WHSC1* was significantly overexpressed in 23 OCCC tissues compared with normal control tissues (*p* = 0.0071; Fig. 1a and b). To confirm protein expression levels of WHSC1 in OCCC tissues, we performed IHC analysis using an antibody of WHSC1. The IHC data showed a strong WHSC1 staining in the nucleus of cancer cells but weak or no staining in the normal tissue. However, no statistical correlation was observed between expression levels and stage (Additional file 1: Table S5). These results suggested that WHSC1 is highly upregulated in OCCC (Fig. 2).

### WHSC1 promotes OCCC cell growth through H3K36 dimethylation

**Fig. 3:**
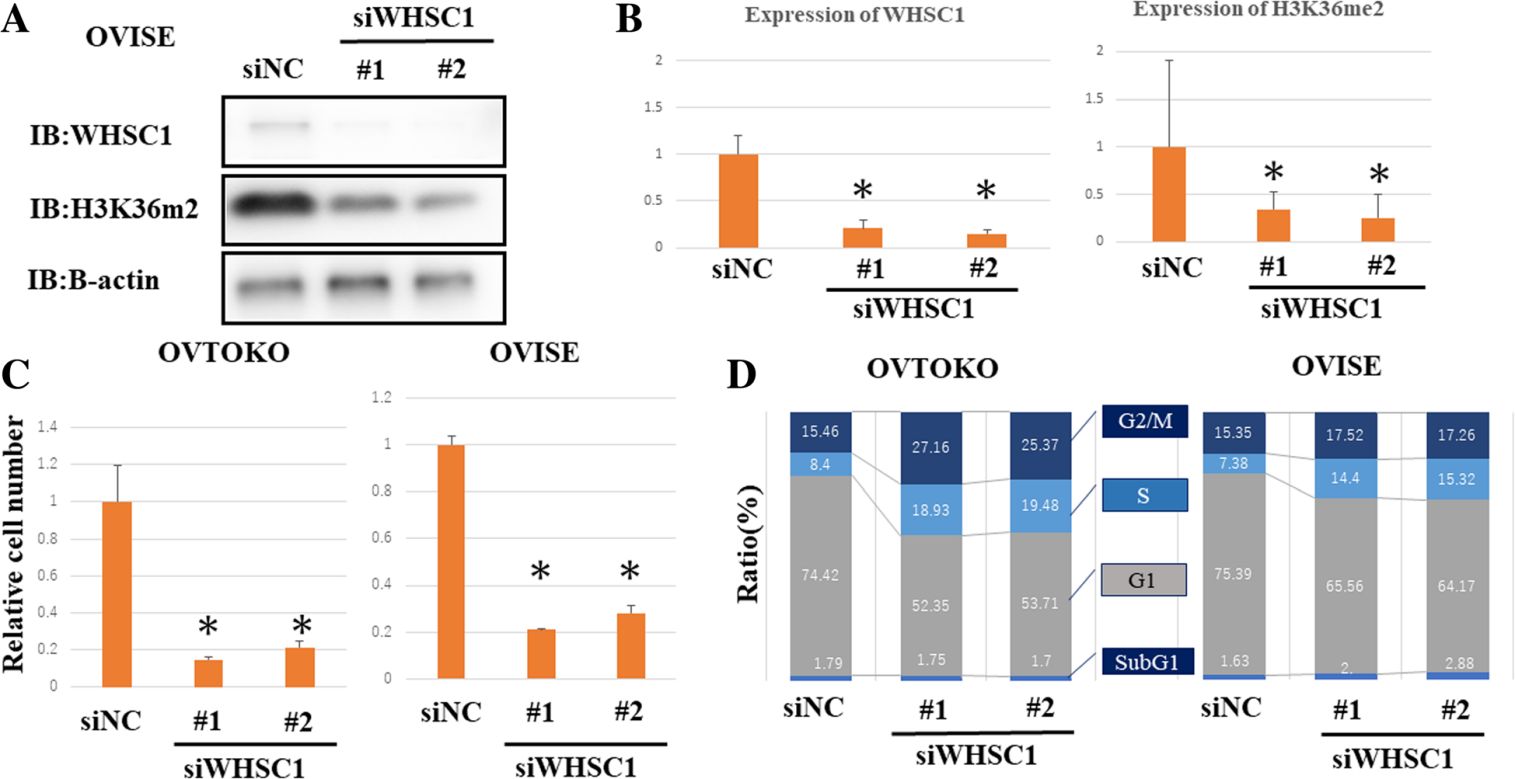
Knockdown of *WHSC1* significantly suppresses cell growth in OCCC cells. **a** Knockdown of *WHSC1* decreased WHSC1 and H3K36me2 levels, as shown by immunoblotting. We transfected OVISE cells with WHSC1-specific siRNAs (siWHSC1#1 and siWHSC1#2) or control siRNA (siNC) for 48 h. Then, immunoblotting was performed for WHSC1, H3K36me2, and *β*-actin. **b** Immunoblot band intensities were quantified using ImageJ. **c** Analysis of cell viability after knockdown of WHSC1 for 72 h in OVTOKO and OVISE cells revealed significant growth suppression. (*p < 0.01). **d** *WHSC1* suppression increased the proportion of S phase cells, as shown by FACS analysis. OVTOKO and OVISE cells were treated with WHSC1-specific siRNAs or siNC, and flow cytometry and PI staining were performed to examine cell cycle status.

To investigate whether WHSC1 overexpression is involved in the growth of OCCC cells, we knocked down the expression of WHSC1 using siRNAs targeting WHSC1 in the OCCC cell lines, with siNC transfection performed separately. We confirmed the knockdown of WHSC1 in the OCCC cell lines by western blotting. Consistent with previous reports, we confirmed decreased levels of H3K36me2 (Fig. 3a and b). Cell-counting assays revealed significant growth suppression in ARID1A mutated OCCC cell lines after WHSC1 knockdown, although no effect was observed for control siRNA and non ARID1A mutated OCCC cells, RMG-ells (Fig. 3c, Additional file 2: Figure S1A, B). To further clarify the mechanism through which WHSC1 knockdown induces growth suppression, we investigated the cell cycle status of OCCC cells by FACS analysis. Cell cycle analysis after WHSC1 knockdown showed an increase in the proportion of cells in the S phase, indicating that WHSC1 knockdown affects cell cycle progression in OCCC cells (Fig. 3d).

### EZH2 regulates WHSC1 expression in OCCC cells

**Fig. 4:**
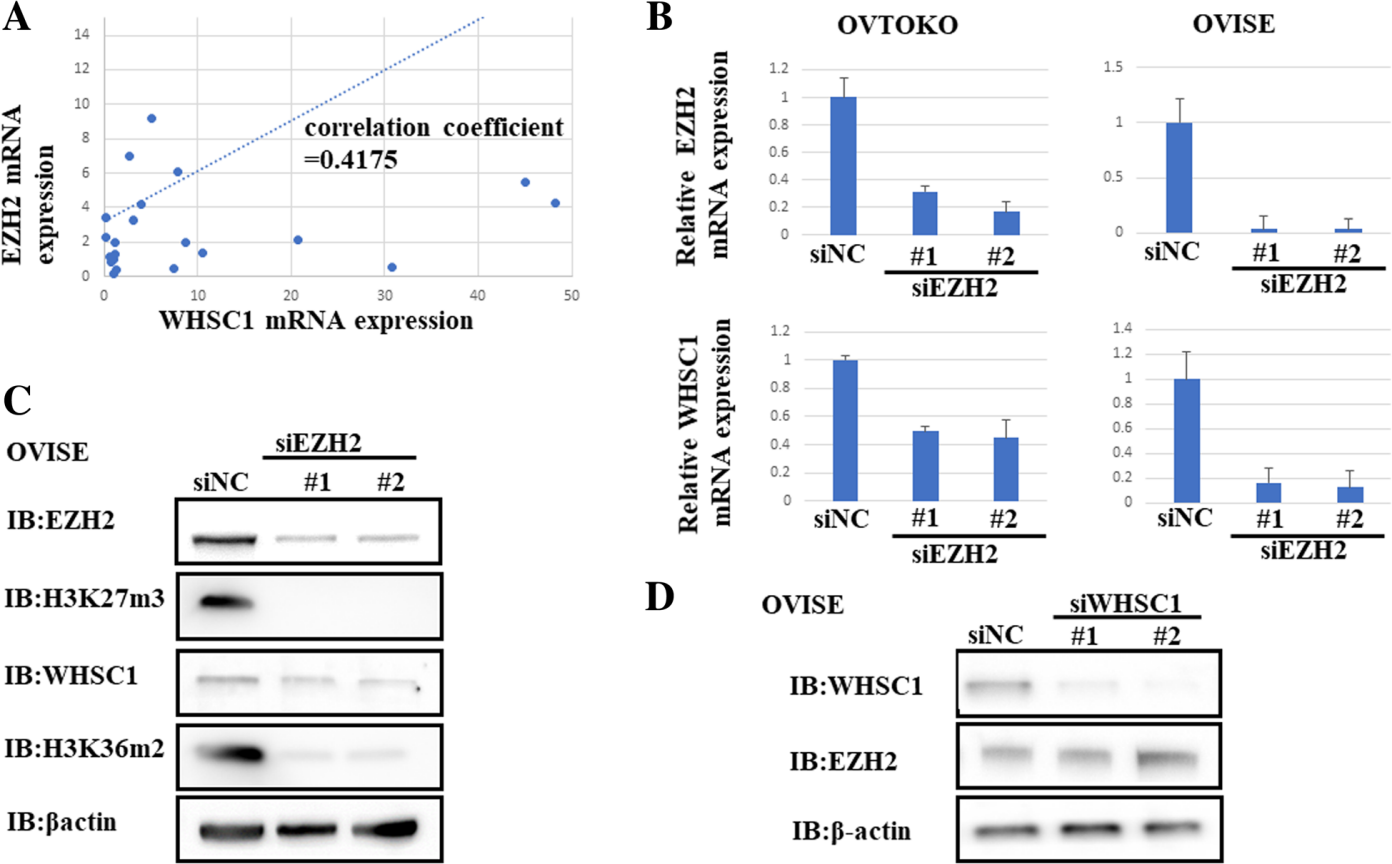
Correlation between *WHSC1* and *EZH2* expression in ovarian clear cell carcinoma. *EZH2* knockdown was found to affect *WHSC1* expression and its associated transcriptional activation marker, H3K36me2. **a** Correlation between the mRNA expression of *WHSC1* and *EZH2* indicating a positive correlation (correlation coefficient = 0.4175). **b** OVTOKO and OVISE cells were transfected with siRNAs (siNC and siEZH2#1 or #2). Knockdown of *EZH2* by siEZH2 was confirmed by qPCR. **c** Expression of EZH2, H3K27me3, WHSC1, H3K36me2, and *β*-actin in OVISE cells with *EZH2* knockdown, as assessed by western blotting. **d** After OVISE cells were transfected with siRNAs (siNC and siWHSC1#1/#2), western blotting was performed. Knockdown of *WHSC1* did not affect the expression of *EZH2*

To investigate the correlation between *WHSC1* and *EZH2* expression in OCCC cells, we analyzed their expression in 23 OCCC by RT-PCR. We found a significant correlation between *WHSC1* and *EZH2* mRNA levels (Fig. 4a). To examine the functional relationship between EZH2 and WHSC1, we knocked down EZH2 using siRNAs (siEZH2 #1, #2) and checked the expression levels of WHSC1 in OCCC cell lines. Interestingly, WHSC1 expression at the mRNA and protein level was significantly decreased with EZH2 knockdown in OCCC cells (Fig. 4b and c).

**Fig. 5:**
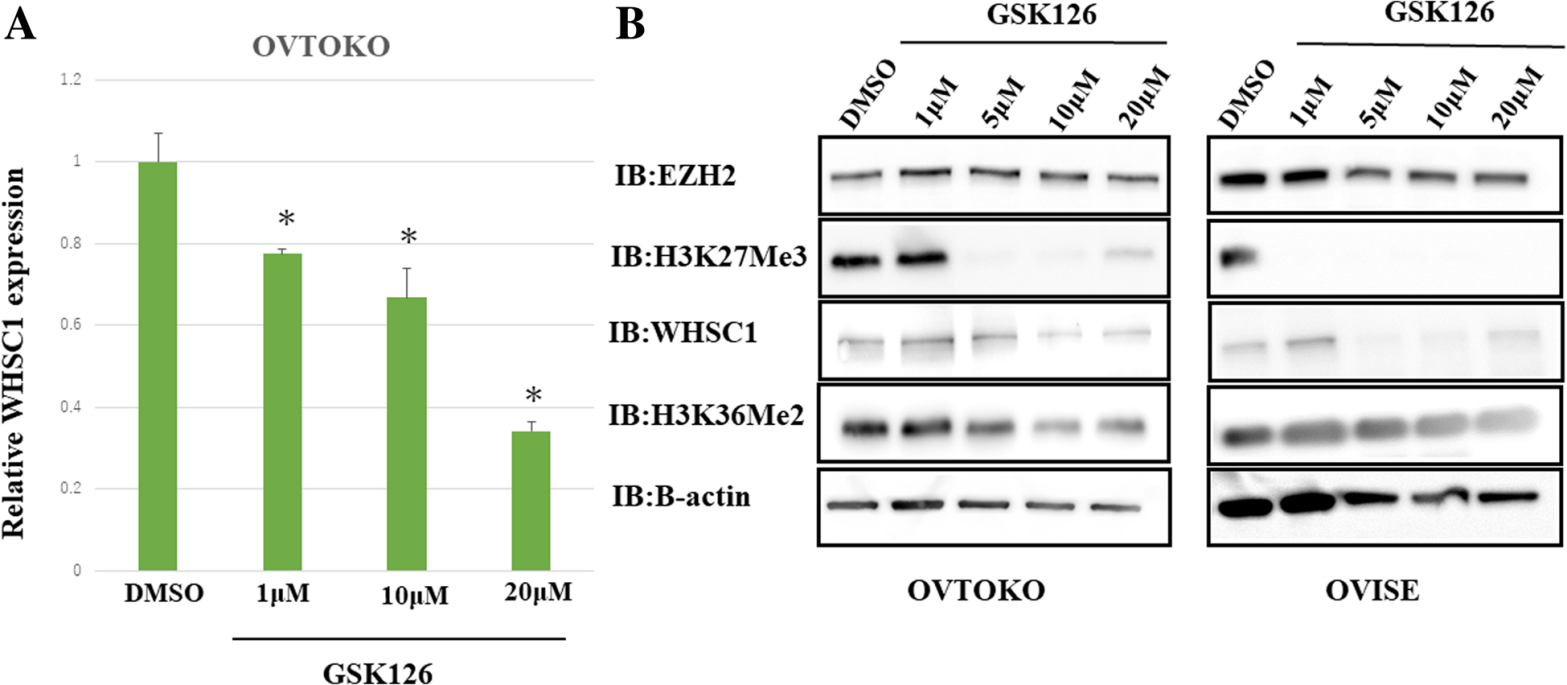
Effect of the EZH2 inhibitor, GSK126, on *WHSC1* expression in OCCC. **a** OVTOKO cells were treated with different concentrations of GSK126 or DMSO for 96 h. qPCR showed a significant decrease in *WHSC1* expression. (*p < 0.01). **b** Western blotting revealed that the protein levels of WHSC1 and H3K36me2 decreased after treatment with GSK126 in a concentration-dependent manner

Consistent with these results, we found reduced levels of H3K36me2 and H3K27me3, which are histone marks catalyzed by WHSC1 and EZH2, respectively (Fig. 4c). However, knockdown of WHSC1 did not change the levels of EZH2 and H3K27me3 (Fig. 4d, Additional file 3: Figure S2). Furthermore, we also studied WHSC1 expression after treatment with the selective EZH2 inhibitor GSK126. We found that WHSC1 expression and H3K36me2 levels decreased in a dose dependent manner after treatment with GSK126, similar to the knockdown of WHSC1 with siRNA (Fig. 5a and b).

## Discussion

In this study, the expression of WHSC1 in OCCC cells was significantly higher than that in normal cells. We showed that WHSC1 overexpression is involved in OCCC cell growth, likely through H3K36 dimethylation. Additionally, we found that suppression of WHSC1 attenuates OCCC cell proliferation and inhibits cell cycle progression. Finally, we also showed that WHSC1 is a downstream gene of EZH2.

There are some reports that have identified WHSC1 overexpression in several cancers types [11, 12] including lung and bladder cancers and hepatocellular carcinoma [21, 22]. In gynecological cancers, WHSC1 was found to be upregulated in ovarian serous carcinoma and endometrial cancers. WHSC1 was overexpressed in about 50% of serous ovarian carcinoma patients, which was found to correlate with poor prognosis [23]. Based on IHC data, WHSC1 was significantly overexpressed in endometrial cancer. In addition, positive WHSC1 expression showed a significant correlation with poorer prognosis [24]. However, there are no reports of the expression profile and function of WHSC1 in OCCC. To the best of our knowledge, our study, involving expression analyses using RT-qPCR and IHC, is the first to show that WHSC1 is significantly overexpressed in OCCC. Although no statistical significance was observed between expression levels and stage (Additional file 1: Table S5), our data suggest that WHSC1 is upregulated at an early stage of OCCC carcinogenesis and remains high in advanced stages of the disease. However, one of the difficulties associated with studies of the expression profiles of OCCC is that normal ovarian tissue may not be a precursor of OCCC because many reports suggested that OCCC is uniquely associated with endometriosis, which is characterized by ectopic endometrial-like epithelium and stroma [4]. Thus, further study about the expression profile of OCCC should be conducted.

Since dimethylation of H3K36 by WHSC1 is sufficient for gene activation, overexpression of WHSC1 could promote cell proliferation likely through the changes in chromatin accessibility mediated by H3K36 dimethylation.

Moreover, there are some reports on the downstream genes that are regulated by WHSC1. For instance, NIMA-related kinase-7 (NEK7) was directly regulated by WHSC1 via H3K36 dimethylation as demonstrated by chromatin immunoprecipitation assays. WHSC1 increase cell proliferation through regulating NEK1 [25]. Cell cycle analysis showed that knockdown of WHSC1 increased the proportion of cells in the S phase. It was previously reported that WHSC1 depletion results in an increased proportion of cells in the S phase. Slower or stalled S-phase progression due to suppression of DNA replication by WHSC1 knockdown could potentially increase the cells in the S-phase [26]. Consistent with their findings, our data suggested that knockdown of WHSC1 could affect the cell cycle. However, in general, G1/S arrest showed a decrease in the proportion of cells in the S phase. In addition, the anti-tumor effect of WHSC1 knockdown was not due to apoptosis because FACS analysis showed that knockdown of WHSC1 did not increase the population of sub-G1 cells. Thus, further analysis to elucidate the mechanism of anti-tumor effect after suppression of WHSC1 will be needed.

Many studies have proven that EZH2 is upregulated in several types of cancers and has anti-cancer therapeutic potential. Several reports showed that some compounds have direct and selective inhibition of EZH2. [27, 28]. GSK126, an EZH2 inhibitor, significantly inhibited cell proliferation of some types of cancers [29]. Moreover, inhibition of the EZH2 methyltransferase was found to induce a synthetic lethality in ARID1A-mutated OCCC cells, and ARID1A mutation status was correlated with sensitivity to an EZH2 inhibitor [15]. In addition, it has been reported that EZH2 is upstream of WHSC1 in several types of cancer cells [30]. It is possible that WHSC1 was regulated by EZH2 through H3K27me3. We used public data (ChIP-Atlas; https://chip-atlas.org) to check which signals of histone modification were increased in the promoter region of WHSC1. We found that, although histone marks for transcription such as, H3K4me3 and H3K27ac were activated in the promoter region of WHSC1, H3K27m3 was not increased. Additionally, in a previous report, the mechanism underlying the upregulation of WHSC1 was induced by EZH2 through suppression of a set of miRNAs that lead to the transcriptional repression of WHSC1 in cancer cells [30].

These data suggested that WHSC1 was indirectly regulated by EZH2. However, in the future, ChIP assay using H3K27m3 to check the promoter region of WHSC1 will improve this study.

In consistence with previous reports, we found that EZH2 regulates WHSC1 expression in OCCC cells, modulating histone H3K36me2, which is associated with transcriptional activation. In addition, GSK126, a selective EZH2 inhibitor suppressed WHSC1 expression in OCCC cells. These results indicate that GSK126 inhibited OCCC cell proliferation by the suppression of H3K36me2 expression via the attenuation of WHSC1 expression. In addition, expression profile analysis by RT-PCR showed a significant correlation between WHSC1 and EZH2 mRNA levels. However, this correlation was weak as well as the *p*-value. Basically, our present data have not elucidated that H3K36me2 was regulated by EZH2 through WHSC1 expression. It is possible that H3K36me was regulated by EZH2 regardless of WHSC1 expression. Further experiment will be needed to clarify this.

It is important to note that this study has some limitations. First, in vivo experiments using cell line-based and patient-derived tumor xenografts may be needed to examine the therapeutic potential of WHSC1 in OCCC. Second, biomarkers for WHSC1 suppression remain to be identified. Our data showed that knockdown of WHSC1 did not decrease cell proliferation in non-ARID1A mutated cells. Thus, we hypothesized that ARID1A mutation status may be involved in an anti-tumor effect of WHSC1 suppression similar to EZH2. However, our data were not enough to confirm this hypothesis and thus further analysis will be required.

## Conclusion

In summary, our results suggested that WHSC1 overexpression leads to cell proliferation in OCCC. Our findings clearly indicate that targeting downstream oncogenic effectors of EZH2 such as WHSC1, which is involved in transcriptional activation, might provide alternative therapeutic strategies. Thus, our data indicated that WHSC1 is a novel therapeutic target against OCCC.

## Additional files

Additional file 1: **Table S1.** Clinicopathological background in 26 patients. **Table S2.** Primer Sequences for Quantitive RT-PCR. **Table S3.** Clinicopathologic characteristics of tissues on IHC. **Table S4.** siRNA Sequences. **Table S5.** Comparison of expression between 2 groups divided by age and stage. (DOCX 18 kb)

Additional file 2: **Figure S1**. Knockdown of WHSC1 did not suppress cell growth in non-ARID1A mutated OCCC cells. (A) Knockdown of WHSC1 decreased WHSC1 levles as shown by immunoblotting. Then, immunoblotting was performed for WHSC1 and β-actin. (B) Analysis of cell viability after knockdown of WHSC1 for 72 h in RMG1 showed that WHSC1 knockdown did not suppress cell growth. (TIF 81 kb)

Additional file 3: **Figure S2.** Knockdown of *WHSC1* did not affect the expression of H3K27me3. After OVOTKO cells were transfected with siRNAs (siNC and siWHSC1#1/#2), western blotting was performed. Knockdown of *WHSC1* did not affect the expression of H3K27me3. (TIF 85 kb)

## Abbreviations

ANOVA: Analysis of variance
ARID1A: AT rich interactive domain 1A
DAB: Diaminobenzidine
DNA: Deoxyribonucleic acid
EZH2: Enhancer of zeste homolog
FACS: Fluorescence activated cell sorting
GAPDH: Glyceraldehyde-3-phosphate dehydrogenase
H3K27me3: Trimethylation of lysine 27 of histone H3
H3K36me2: Dimethylation of lysine 36 of histone H3
IHC: Immunohistochemistry
MMSET: Multiple myeloma SET domain
MTT assays: 3-[4,5-dimethylthiazol-2-yl]-2,5-diphenyltetrazolium bromide assays
NEK7: Never-in-mitosis A -related kinase 7
NSD2: Nuclear SET domain-containing 2
OCCC: Ovarian clear cell carcinoma
PBS: Phosphate buffered saline
PCR: Polymerase chain reaction
siNC: Negative control siRNA
siRNA: Small interfering ribonucleic acid
SUV39H2: Suppressor of variegation 3–9 homolog 2
WHSC1: Wolf-Hirschhorn syndrome candidate gene-1
WST: Water Soluble Tetrazorium salts
