# Supplementary material for: The histone methyltransferase WHSC1 is regulated by EZH2 and is important for ovarian clear cell carcinoma cell proliferation
Source: BMC Cancer. 2019 May 15;19:455. doi: 10.1186/s12885-019-5638-9 (PMC6521555; doi:10.1186/s12885-019-5638-9)
Supplement: Supplementary file 1 — Table S1. Clinicopathological background in 26 patients. Table S2. Primer Sequences for Quantitive RT-PCR. Table S3. Clinicopathologic characteristics of tissues on IHC. Table S4. siRNA Sequences. Table S5. Comparison of expression between 2 groups divided by age and stage. (DOCX 18 kb) [file 12885_2019_5638_MOESM1_ESM.docx]

Table1. Clinicopathological background in 26 patients

| No. | Age(years) | Histologic Diagnosis | stage(TNM) |
| --- | --- | --- | --- |
| 1 | 60-69 | clear cell carcinoma | pT1c1N0M0 |
| 2 | 50-59 | clear cell carcinoma | pT3cN0M0 |
| 3 | 50-59 | clear cell carcinoma | pT1c3N0M0 |
| 4 | 70-79 | clear cell carcinoma | pT1c2N0M0 |
| 5 | 40-49 | clear cell carcinoma | pT3cN0M0 |
| 6 | 40-49 | clear cell carcinoma | pT3bN1M0 |
| 7 | 50-59 | clear cell carcinoma | pT1c3N0M0 |
| 8 | 40-49 | clear cell carcinoma | pT1c1N0M0 |
| 9 | 40-49 | clear cell carcinoma | pT1c1N0M0 |
| 10 | 50-59 | clear cell carcinoma | pT1aN0M0 |
| 11 | 40-49 | clear cell carcinoma | pT3bN1M0 |
| 12 | 70-79 | clear cell carcinoma | pT1c2N0M0 |
| 13 | 50-59 | clear cell carcinoma | pT3bN1M1 |
| 14 | 50-59 | clear cell carcinoma | pT1c3N0M1 |
| 15 | 60-69 | clear cell carcinoma | pT1c1N0M0 |
| 16 | 50-59 | clear cell carcinoma | pT1bN0M0 |
| 17 | 60-69 | clear cell carcinoma | pT1c3N0M0 |
| 18 | 50-59 | clear cell carcinoma | pT1c3N0M0 |
| 18 | 50-59 | clear cell carcinoma | pT2aN0M0 |
| 20 | 50-59 | clear cell carcinoma | pT1aN0M0 |
| 21 | 30-39 | clear cell carcinoma | pT1c3N×M０ |
| 22 | 60-69 | clear cell carcinoma | pT1c2N×M0 |
| 23 | 70-79 | clear cell carcinoma | pT3cN0M0 |
| N1 | 30-39 | normal |  |
| N2 | 60-69 | normal |  |
| N3 | 50-59 | normal |  |

Table2. Primer Sequences for Quantitive RT-PCR

| Gene Name | |  |  | Primer Sequence |  |
| --- | --- | --- | --- | --- | --- |
| GAPDH (housekeeping gene)-f | | |  | 5’-GAAGGTGAAGGTCGGAGTC-3’ | |
| GAPDH (housekeeping gene)-r | | |  | 5’-GAAGATGGTGATGGGATTTC-3’ | |
| WHSC1-f |  |  |  | 5’-TCGAAGCAGCTCTTGTGTCTAAG -3’ | |
| WHSC1-r |  |  |  | 5’-TTTGGACCACACCAAATCACCAAC -3’ | |
| EZH2-f |  |  |  | 5’-CGCTTTTCTGTAGGCGATGT-3’ | |
| EZH2-r |  |  |  | 5’-TGGGTGTTGCATGAAAAGAA-3’ | |

Table3. Clinicopathologic characteristics of tissues on IHC

| Age(years) | Histologic Diagnosis | stage(TNM) |
| --- | --- | --- |
| 50-59 | clear cell carcinoma | pT3cN0M0 |
| 40-49 | clear cell carcinoma | pT1c1N0M0 |
| 50-59 | clear cell carcinoma | pT1aN0M0 |
| 40-49 | clear cell carcinoma | pT3bN1M0 |
| 30-39 | normal |  |
| 60-69 | normal |  |

Table 4. siRNA Sequences

| siRNA Name | | Sequence |  |  |  |  |
| --- | --- | --- | --- | --- | --- | --- |
| siWHSC1 #1 | | Sense: | 5’-CAGAUCUACACAGCGGAUA -3’ | | | |
|  |  | Antisense: | 5’-UAUCCGCUGUGUAGAUCUG -3’ | | | |
| siWHSC1 #2 | | Sense: | 5’-GUUAAUUGGCAUAUGGAAU-3’ | | | |
|  |  | Antisense: | 5’-AUUCCAUAUGCCAAUUAAC-3’ | | | |
| siEZH2#1 |  | Sense: | 5’-CUAACCAUGUUUACAACUA-3’ | | | |
|  |  | Antisense: | 5’-UAGUUGUAAACAUGGUUAG-3’ | | | |
| siEZH2#2 |  | Sense: | 5’-GACAGAAGAGGGAAAGUGU-3’ | | | |
|  |  | Antisense: | 5’-ACACUUUCCCUCUUCUGUC-3’ | | | |

Table5. Comparison of expression between 2 groups divided by age and stage

| Relative expression level | ≧Median | <Median | *P* value |
| --- | --- | --- | --- |
| age | 53.91666667 | 57.72727273 | 0.7136 |
| stage | Ⅰ:8 Ⅲ:3 Ⅳ:1 | Ⅰ:7 Ⅱ:1 Ⅲ:2 Ⅳ:1 | 0.581 |
