# Supplementary figures and images for: The histone methyltransferase WHSC1 is regulated by EZH2 and is important for ovarian clear cell carcinoma cell proliferation
Source: BMC Cancer. 2019 May 15;19:455. doi: 10.1186/s12885-019-5638-9 (PMC6521555; doi:10.1186/s12885-019-5638-9)

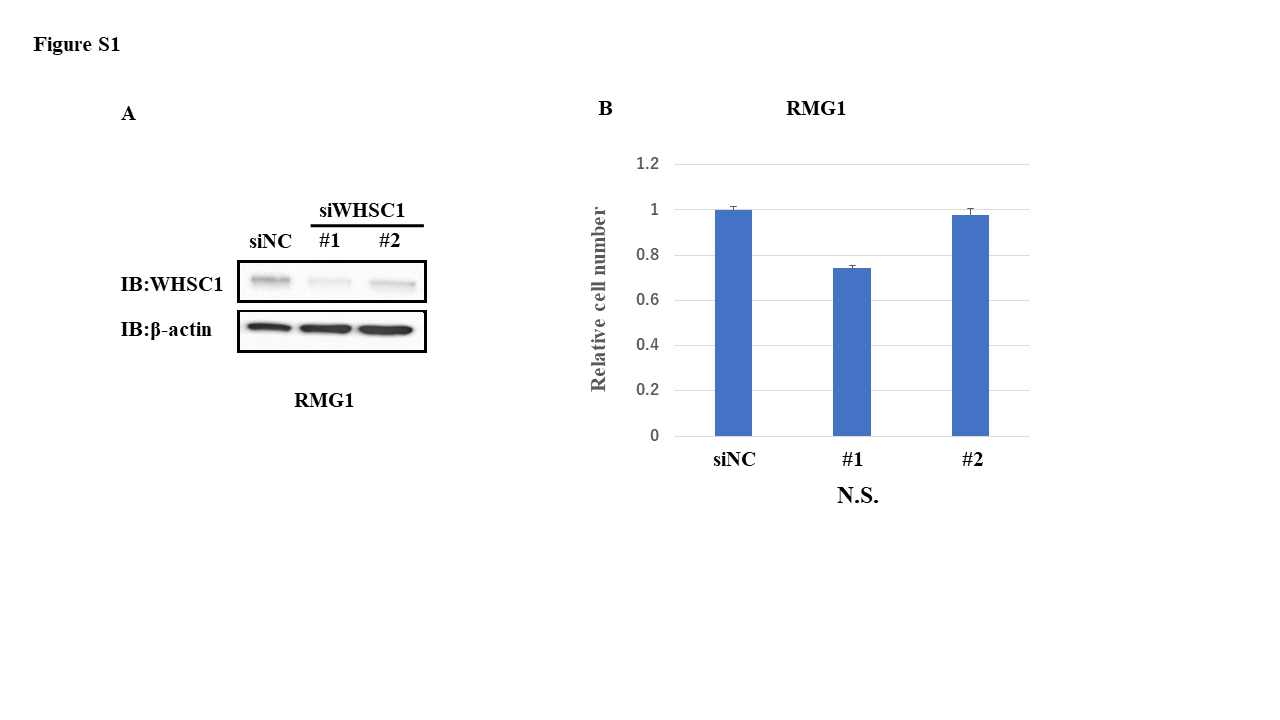

Supplement: Supplementary file 2 — Figure S1. Knockdown of WHSC1 did not suppress cell growth in non-ARID1A mutated OCCC cells. (A) Knockdown of WHSC1 decreased WHSC1 levles as shown by immunoblotting. Then, immunoblotting was performed for WHSC1 and β-actin. (B) Analysis of cell viability after knockdown of WHSC1 for 72 h in RMG1 showed that WHSC1 knockdown did not suppress cell growth. (TIF 81 kb) [file 12885_2019_5638_MOESM2_ESM.tif]

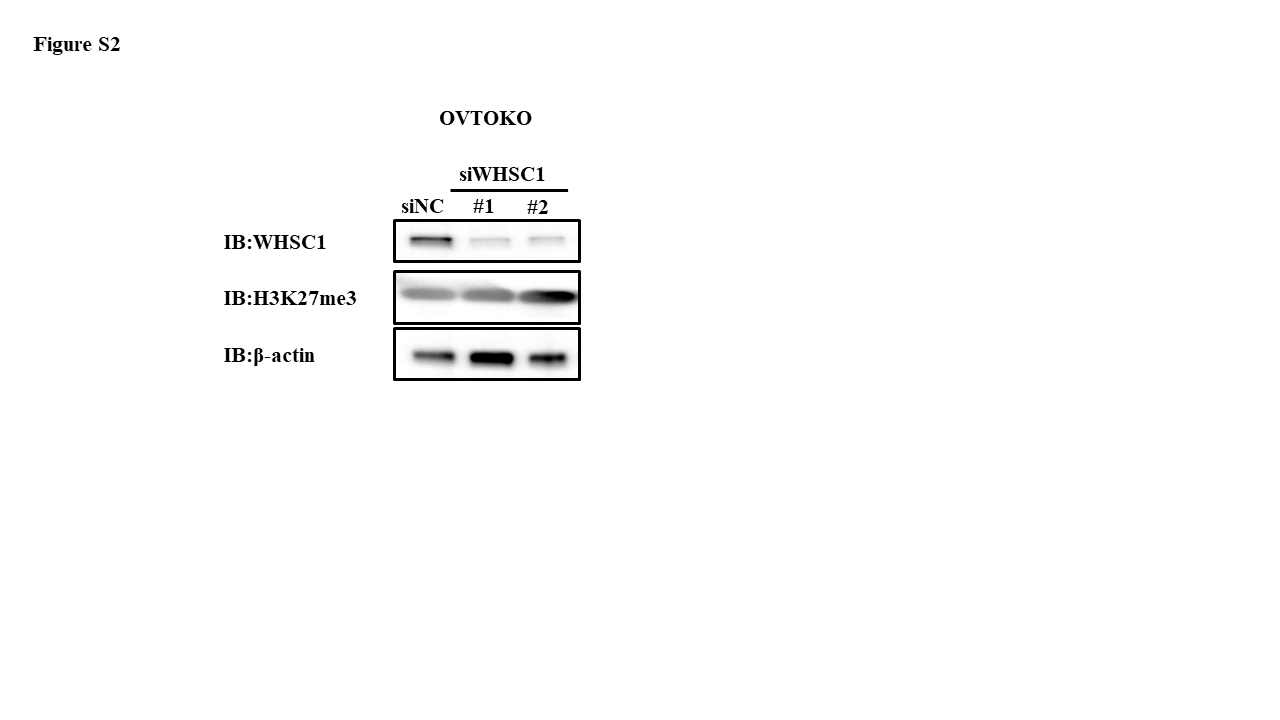

Supplement: Supplementary file 3 — Figure S2. Knockdown of WHSC1 did not affect the expression of H3K27me3. After OVOTKO cells were transfected with siRNAs (siNC and siWHSC1#1/#2), western blotting was performed. Knockdown of WHSC1 did not affect the expression of H3K27me3. (TIF 85 kb) [file 12885_2019_5638_MOESM3_ESM.tif]
